# Study protocol: evaluation of a community health promotion program in a socioeconomically deprived city district in the Netherlands using mixed methods and guided by action research

**DOI:** 10.1186/s12889-019-6389-x

**Published:** 2019-01-16

**Authors:** Marja A. J. G. de Jong, Annemarie Wagemakers, Maria A. Koelen

**Affiliations:** 1 GGD IJsselland (Municipal Health Service), Postbox 1453, 8001 BL Zwolle, The Netherlands; 2 Health & Society, Department of Social Sciences, Wageningen University & Research, P.O. Box 8130, 6700 EW Wageningen, The Netherlands

**Keywords:** Community health promotion, Citizen participation, Intersectoral collaboration, Health supportive environments, Action research

## Abstract

**Background:**

Voorstad on the Move (VoM) is a community health promotion program implemented in a socioeconomically deprived city district in the Netherlands. Based on exploration of the health situation, concurrent views on health promotion, and insights from literature, VoM is grounded in a social-ecological perspective and puts three action principles center core: citizens’ participation, intersectoral collaboration, and a health supportive environment. VoM aims to improve the health of inhabitants, mostly low socioeconomic status (SES) families, and to realize changes in the social and physical environment. This current research, as part of the wider VoM project, aims to study the impacts and action principles of VoM. The main research questions concern the inhabitants’ perceptions on health and health supportive environments, the perceived benefits of citizen participation in terms of health literacy and empowerment, and the factors and mechanisms that contribute to citizen participation and intersectoral collaboration.

**Methods:**

The study has a mixed methods design, including process evaluation and monitoring, and combines qualitative and quantitative data. Research activities include literature study, in-depth interviews, focus group discussions, concept and capacity mapping, document analysis, and health survey data. A prominent strategy is action research, which aims to involve all stakeholders, capturing the different perspectives of citizens and professionals, and engaging low SES groups. The principle of triangulation is continuously applied to optimize the reliability of this study, using multiple methods and multiple sources. Internal validity is enhanced by triangulation of methods and resources. Other verification techniques will also be used, such as expert consultation.

**Discussion:**

The design of the study, with a strong focus on action research, facilitates the involvement of all stakeholders and contributes to the development of capacities, learning, and empowerment, and thus contributes to health. The VoM program is innovative because it adopts an open approach in which activities evolve from citizens’ needs, with a focus on action elements. This study will unravel the mechanisms of the action elements at community level, thereby helping to find ways to reduce health inequities. The findings will further elucidate what works and why it works for low SES groups.

## Background

In the Netherlands, less educated inhabitants live – on average – six years less than most educated people, and the difference in healthy life expectancy between these groups is almost 19 years [1]. Although the healthy life expectancy of less educated people has increased considerably in the last decade, the difference in life expectancy between the two groups has remained the same [1].

Health inequities are a complex problem caused by the interplay between individuals, groups, communities, and multiple factors in the social, physical, and economic environment [2–5]. To date, health promotion programs have not been successful in substantially reducing the health gap between the higher and the lower socioeconomic groups. It is therefore a challenge to develop more effective strategies [6–10]. These strategies should be based on an ecological perspective, addressing factors at multiple levels and looking at the interaction between factors [11–13].

Such strategies are being developed in the community health promotion program called Voorstad on the Move (VoM). In line with national and local policy objectives, the aim of the program is to contribute to the improvement of health and to find ways to reduce health inequities [14, 15]. The program is being implemented in four socioeconomically deprived neighborhoods in a city district of 10,750 inhabitants in a city in the east of the Netherlands between July 2016 and January 2020. In Voorstad, both the socioeconomic status (SES) and the health status of inhabitants are relatively low compared with other parts of city [16].

### Casus: Community health promotion program ‘Voorstad on the Move’ (VoM)

VoM is grounded in a social-ecological perspective, based on the exploration of the health situation in Voorstad, concurrent with views on health promotion and insights from the literature [17–21]. VoM puts three action principles at its center: citizen participation, intersectoral collaboration, and a health supportive environment, that that were emanated from the results of an preparatory study (Oct–Dec 2015) [22].

The aim of the preparatory study was to get an impression of the health situation in Voorstad in order to decide on the program goals and methods. This preparatory study consisted of seven focus groups with citizens (*n* = 40) and 30 interviews with professionals from different disciplines about health and health behaviors. Overall, the top three most mentioned aspects of health were: feeling at ease (no stress), being in control, and being together with friends, family, and neighbors (joint activities). There was a clear focus on health as an asset or resource for meaningful living [23, 24]. The inhabitants barely mentioned unhealthy lifestyles, e.g. obesity and smoking, which are the focus of professionals and of the data in monitor and health surveys. These qualitative data were supplemented with quantitative data from health monitors [25] and the local citizens’ survey [26]. The discrepancy in perceptions on health and wellbeing between inhabitants and professionals emphasizes the need to include citizens’ perceptions in health promotion activities [19, 20, 27].

The Voorstad inhabitants’ viewpoints are the starting point for VoM, thereby giving the inhabitants ownership to address health in a positive way, focusing on assets and resources [24]. This means that activities in the program are not chosen or planned beforehand, but rather developed and implemented as a result of questions and needs expressed by Voorstad inhabitants. Citizens’ active involvement and responsibility for activities strengthen their health literacy and empowerment [28–30]. Citizen participation, including defining ‘health’, developing, implementing, and evaluating activities with and by the citizens [31, 32], is one of the action principles in VoM.

Another important finding of the preparatory study was the presence of a comprehensive infrastructure of public, welfare, social support, sports and care organizations, community centers, and (informal) networks and alliances in which both professionals and inhabitants collaborate [22]. Intersectoral collaboration [33–36] between primary care, social services, and environmental, policy, and public health workers is therefore a second action principle of the program. VoM joins and uses the existing social infrastructure to add the broader view on health and bring in knowledge to make health promotion activities possible.

Both inhabitants and professionals mentioned barriers that hinder healthy living and keep them from changing behavior, such as accessibility of sports facilities and prices of healthy foods, as well as social norms, attitudes, and habits. This indicates the third action principle of VoM: creating a supportive social and physical environment for health [37–39].These action principles can be defined as actions, processes, or mechanisms that help establish the effect or impacts of a health promotion program [40–42]. The premise of principles for action is that they contribute to health through multiple pathways and serve multiple purposes, such as program effectiveness, the creation of supportive environments for health, and empowerment of all stakeholders, both professionals and citizens [32, 43].

In July 2016, two health brokers started to support these action principles by facilitating citizens’ participation in developing and implementing activities that fit citizens’ needs and build healthy alliances. Recent studies show that the broker role is essential in facilitating intersectoral collaboration and exchanging knowledge between stakeholders [44–46].

The aim of the VoM program is to improve the perceived health of the Voorstad inhabitants, mostly low SES families, and achieve changes in the social and physical environment that support health and healthy behavior. The overall research aim is to study the impacts and action principles of VoM comprehensively on different levels. This will contribute to finding ways to reduce existing health inequities. Therefore, four interrelated research questions (RQs) have been formulated:

1. How do Voorstad inhabitants perceive health and health supportive environments?
2. What benefits do citizens who participate in the Voorstad on the Move program observe in terms of perceived health, health literacy, and empowerment?
3. What factors and mechanisms contribute to citizen participation and intersectoral collaboration?
4. What is the overall impact of the Voorstad on the Move program in terms of health promotion activities, social and physical environment, and inhabitants’ perceived health?

## Theoretical framework

Because the VoM program is based on a social-ecological perspective on health, the theoretical framework consists of different theories and models that recognize the link between practice and context within social situations.

**Fig. 1:**
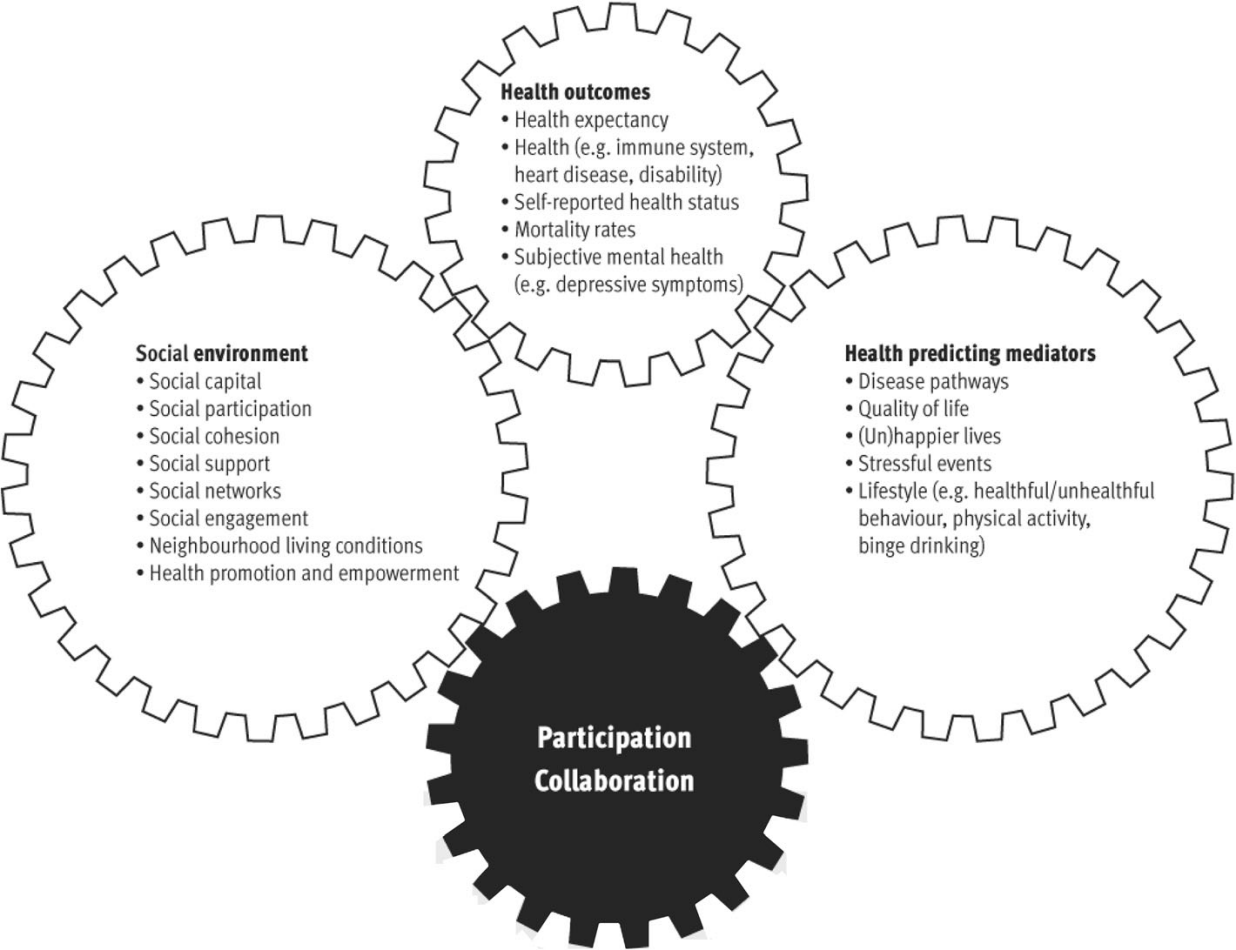
Framework to facilitate and evaluate community health promotion [48]

To study and understand impact on health and the environment on the one hand and the working of the action principles on the other hand, a framework to facilitate and evaluate a community health promotion program will be used [32, 47, 48]. This framework [Fig. 1] visualizes the relation between the social environment, health predicting mediators (e.g. lifestyle), and population health status (e.g. perceived health). It provides operationalizable variables that moderate the relation between the social environment and health predicting mediators. The moderating variables are the action principles in the VoM program. Citizen participation, intersectoral collaboration, and a health supportive environment are used as entry points to make the social environment of health researchable and manageable by communities.

Social practice theory (SPT) [49], the reasonable person model (RPM) [50], and the Healthy Alliances (HALL) framework [33] are used to understand the working of the action principles comprehensively and on different levels.

**Fig. 2:**
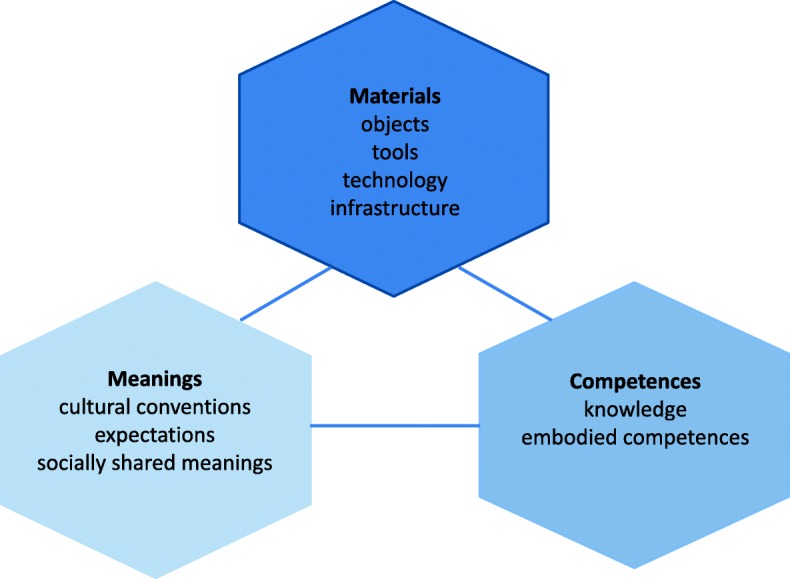
The elements of a social practice. Adapted based on Shove et al. [51]

SPT integrates the individual with his or her social environment and will be used to study the mechanisms of citizen participation and health behaviors. In contemporary theories of social practice, health and wellbeing are considered to be outcomes of participation in a set of social practices, commonly created by the reality of everyday life [49]. Following Shove et al. [51], a practice is defined as being constituted by meanings about how and why to do things (cultural conventions, expectations, and socially shared meanings), materials (objects, tools, and infrastructures), and competences both tacit and explicit (knowledge and embodied skills) [Fig. 2]. In this study, participation and health behaviors will be regarded as social practices rather than only individual behavior, because they fit with the community approach focusing on social change, instead of attempting to change what Shove et al. [51] refer to as individuals’ ABC (attitudes, behaviors, choices).

The RPM is a conceptual framework that links environmental factors with human behavior [50]. People are more reasonable, cooperative, helpful, and satisfied when the environment supports their basic informational needs. The same environmental supports are important factors in enhancing human health. Reasonableness is used, rather than well-being, because it focuses on bringing out the best in people. Central in the RPM is the management of information, either visual or written, indicating that people are more reasonable when their informational needs are met [52].

**Fig. 3:**
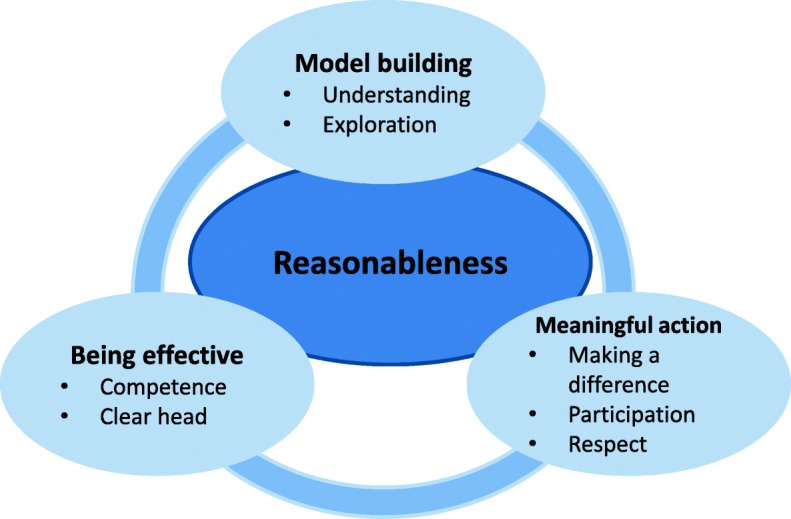
The Reasonable Person Model [52]

The RPM consists of three domains: building mental models, meaningful action, and being effective [Fig. 3]. Mental models influence our perception of what is going on and guide our actions. Meaningful action implies that people feel listened to and respected, even if their wishes are not met. The sense that one is making a difference can go a long way towards bringing out the best in one [53]. Being effective concerns effectiveness and reasonableness, because of mental fatigue. It is about a particular aspect of mental functioning described as directed attention, caused by the many complex and competing demands in one’s environment. The RPM framework will be used to study the way in which the physical environment can be health supportive to the inhabitants. Both SPT and RPM put great importance on the interaction between the environment and the behavior of an individual. They are complementary, as SPT focuses on the social environment and RPM on the natural (physical) environment.

**Fig. 4:**
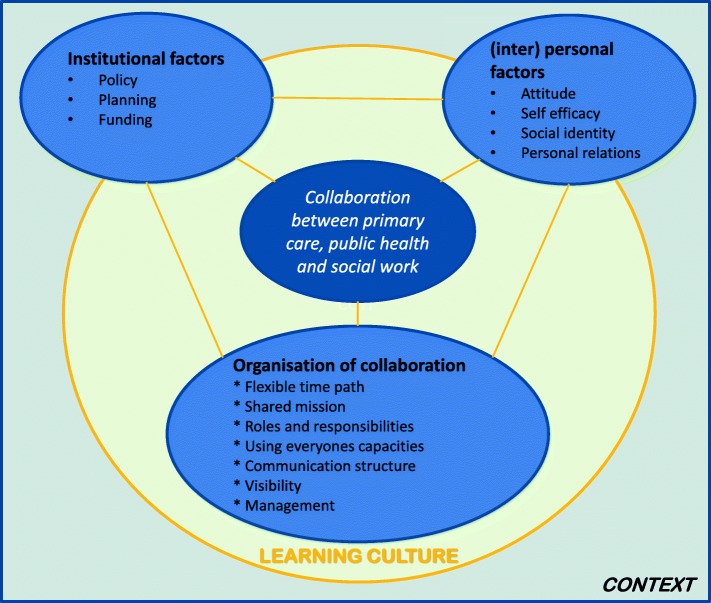
The updated Healthy Alliance framework [54]. Translated from Tol et al., [54]

Within the extended network that exists in Voorstad, intersectoral collaboration is facilitated by a small steering group of partners from the healthcare and societal sectors, with health brokers as essential participants. This group can be regarded as the healthy alliance. The updated HALL framework, will be used to study intersectoral collaboration within the healthy alliance in VoM [Fig. 4]. This framework recognizes three groups of factors – institutional factors, (inter)personal factors, and the organization of the alliance – that can either facilitate or hamper the collaboration between the partners in the alliance [33]. The updated HALL framework visualizes the importance of context and learning culture in intersectoral collaboration [54].

## Methods/design

### Study design

The study will use a mixed-methods design and will combine qualitative and quantitative data. The research activities will include literature study, in-depth interviews, focus group discussions, concept and capacity mapping, document analysis, and analysis of citizens’ survey data [Table 1].

**Table 1:** Study overview – frameworks, methods, tools, participants, and repeats

Research question	Framework	Methods	Tools	Participants	Repeats
*RQ1.* *Perceptions*	SPT RPM	Interviews Photography	Focus groups Photovoice [67, 68]	100 inhabitants 32–40 inhabitants	1 2
*RQ2.* *Participation*	SPT	Literature study Interviews Questionnaire Document analysis	Pretty’s participation ladder [34] Empowerment checklist [74] Health literacy questionnaire (HLS-EU-Q) [71]	100 inhabitants	2
*RQ3.* *Mechanisms*	HALL Framework	Document analysis Interviews Checklist	Coordinated action checklist [48] Participatory network mapping tool (PNMT) [60] Network analysis tool [60]	12 professionals 6–8 network partners	3 3
*RQ4.* *Overall impact*	Logic Model	Literature study Questionnaire Interviews Document analysis	Activities database Photovoice [67, 68] Citizens’ survey (2 yearly) [16] Health monitor (4 yearly) [76]	32–40 inhabitants Representative sample of 600 inhabitants	1 2 3

*Abbreviations: HALL* Healthy Alliance, *HLQ* Health Literacy Questionnaire, *RPM* Reasonable Person Model, *PNMT* Participatory network mapping tool, *RQ* Research Question, *SES* Socioeconomic Status, *SPT* Social Practice Theory, *VoM* Voorstad on the Move

The use of multiple strategies and multiple research methods across multiple levels is assumed to be the most effective approach. The combination of information from multiple sources and methods – triangulation –increases data validity [55]. Also, partners and citizens will be involved in the planning of the research as well as in different research activities.

A prominent strategy is action research, which aims to involve all stakeholders, capturing the different perspectives of citizens and professionals and engaging citizens with low SES. The value of action research is that it reflects the values of health promotion, such as participation and empowerment [6, 56–58]. It thereby facilitates the development of capacities, learning, and empowerment [4] and thus contributes to health [59]. It also enables those involved to continually optimize their strategies [60–62], and it contributes to developing both theories and research methods to understand and explain what works and why it works.

**Fig. 5:**
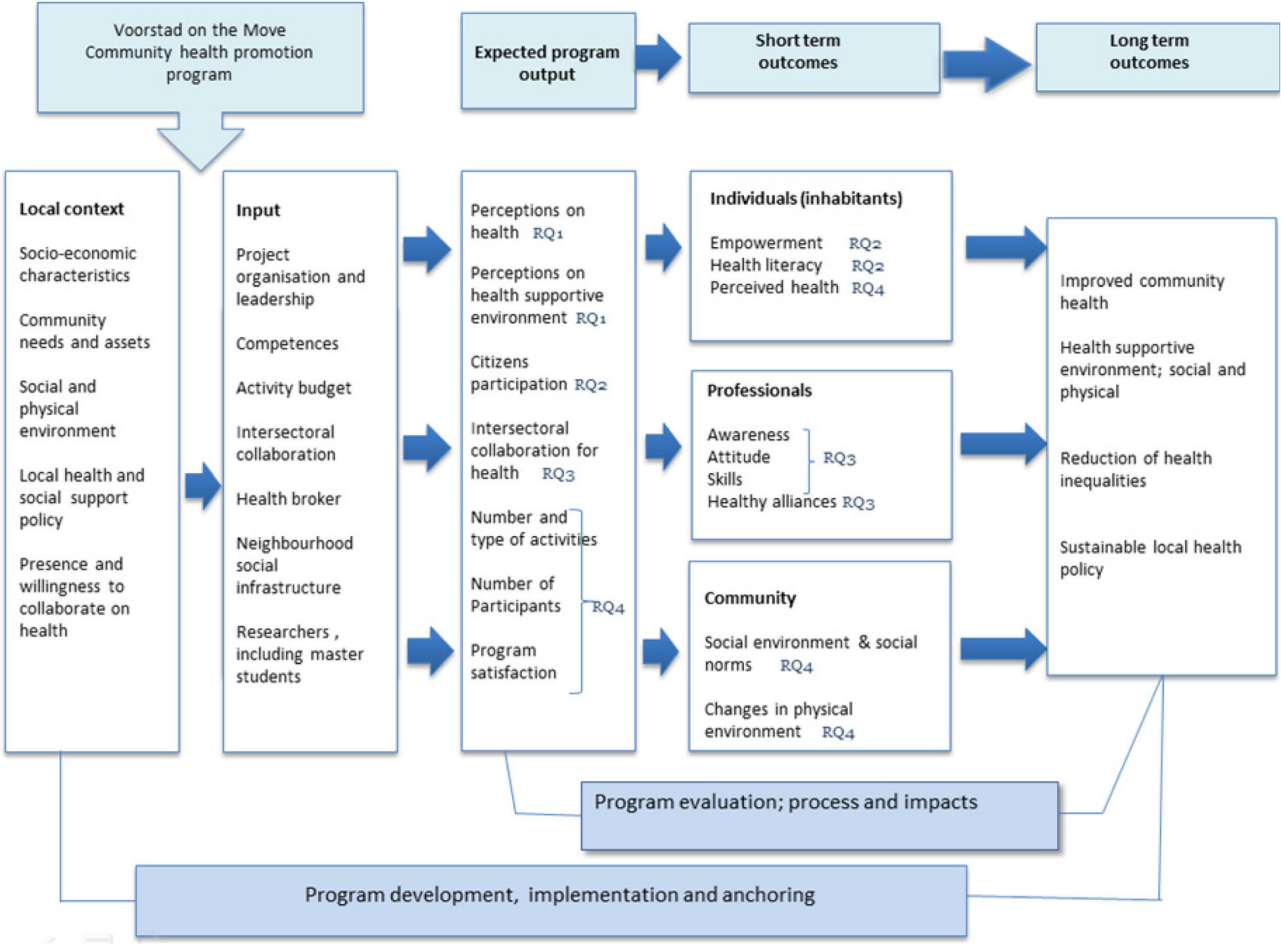
Logic model used for evaluation of Voorstad on the Move. Adapted based on Saan & Haes [63]

To operationalize, and to provide insights into, factors relevant to addressing the RQs, the logic model, based on the framework for planning, implementation, and evaluation of health promotion programs [63], will be used [Fig. 5]. This logic model will help to make explicit the hypothesized pathways; to define processes, output, and outcome indicators at different levels (individual, professional, and community); and to unravel action elements [32, 64].

Figure 5 illustrates the logic model for the impact evaluation of VoM, based on literature on community-based approaches [6, 32, 64] and evaluation studies of complex community health promotion programs [64]. The hypothesis is that a community-based participatory approach to developing and implementing health activities at different levels such as individuals, professionals, and community will result in improved perceived health, a health supportive environment, and sustainable local health policy, leading to a reduction in health inequities in the long term. These long-term expected outcomes will be preceded by measurable short-term outcomes like e.g. health literacy, healthy alliances, and changes in the physical environment, moderated by the action principles. In this model, citizen participation, intersectoral collaboration, and a health supportive environment are defined as program outcomes and, at the same time, are action principles in this principle-based health promotion program [32]*.* The operationalization of each of the four research questions is now set out.

#### Research question 1. How do Voorstad inhabitants perceive health and a health supportive environment?

Perceptions on health and health supportive environments will be measured using focus groups and photovoice.

- Collecting and discussing the perceptions and priorities of the inhabitants about health is the starting point for citizen participation in the VoM program. At the start of the program, focus groups will be held with 15 existing groups of inhabitants to explore perceptions and meanings about health [65, 66]. Results of the first focus group session will be fed back to, and discussed, with the same group in a second session. Participants are challenged to think of actions and plans to work on their own health. In total, about 100 inhabitants will participate in this research study.
- Photovoice will be used to reveal inhabitants’ perceptions of their neighborhood as a source of health opportunities or barriers [67–69]. In total, 32–40 inhabitants (8–10 from each of the four neighborhoods) will be asked to tell ‘the story of the photo or image’.

#### Research question 2. What benefits do citizens who participate in the Voorstad on the move program observe in terms of perceived health, health literacy, and empowerment?

Active participation in health promotion activities, varying from consultation and collaboration to partnership or ownership, can either result from the focus groups or otherwise be initiated by the VoM health brokers or collaborating partners. A total of 100 inhabitants who either participate in focus groups (RQ 1) or are involved in community activities will be ‘followed’ during the program. They will be asked about their way and level of participation using Pretty’s participation ladder [34, 70], health literacy using the 9-item Health Literacy Questionnaire (HLQ) [71–73], and empowerment using the Netherlands Empowerment Checklist [74, 75]. Perceived health is assessed based on the question on self-perceived health: ‘How is your health in general’, which contains five answering categories; 1) very good, 2) good, 3) fair, 4) bad, and, 5) very bad. This question is part of the citizens’ survey which takes place every two years in Deventer [16] and of the Health Monitor conducted by the Municipal Health Services in the Netherlands [76, 77]. Subsequently, in-depth interviews and focus groups will be held to discuss citizens’ perceptions on the connection between participation, perceived health, and empowerment.

#### Research question 3. What factors and mechanisms contribute to citizen participation and intersectoral collaboration?

The HALL framework will be used to study the intersectoral collaboration and active involvement of stakeholders and to identify conditions that contribute to the collaboration and make these alliances successful [33, 54] [Fig. 4]. A special focus will be placed on the role of health brokers, as these seem to be crucial for connecting different sectors [78]. The Coordinated Action Checklist [48] will be used to evaluate and facilitate the collaboration of the core stakeholders, members of the Voorstad social team, the neighborhood manager, health brokers, and the program coordinator. The results of the checklist on various dimensions, such as task, relations, growth, and visibility, will be discussed with this core group. These evaluation sessions will be held once a year, in total three times.

A document analysis of all the reports, plans, and notes produced by the project team will be used to describe the collaboration processes that have taken place. Furthermore, a network analysis [35, 60] will be conducted to map the collaborating organizations – community centers, schools, grassroots organizations, and neighborhood sports club – that take part in the program irregularly and on a less structured basis. Five to 10 organizations will be interviewed twice (2018 and 2020) to get insights into the impact of VoM, the collaboration processes [79], and the health broker role.

#### Research question 4. What is the overall impact of the Voorstad on the move program in terms of health promotion activities, social and physical environment, and inhabitants’ perceived health?

Results and outcomes of the program will be measured on different levels: individual, professional, and community [Fig. 5]. At the individual level, perceived health, lifestyle, and health behaviors have been or will be measured in the local citizens’ survey every two years (2015: T0; 2017: T1; 2019: T2) [16]. Additionally, the health monitors [76] carried out in 2016 and 2020 will provide more detailed information on the health status of the city district, Voorstad. In both surveys, citizens’ health and lifestyle data are monitored at neighborhood level.

The program activities are monitored in a so-called activity database. The number and type of health promotion activities developed with citizens’ involvement and the number of participants per activity will be registered, thereby monitoring the program output. Citizens who participate in the program activities will be asked about their satisfaction. In order to map changes in perceptions of the social and physical environment, the photovoice study (RQ1) will be repeated in 2019.

Qualitative research data from interviews and focus group discussions will be audiotaped, transcribed, and analyzed using Atlas-ti to manage the data and guarantee transparency. A coding scheme based on theory and the framework will be developed to analyze the qualitative data stepwise, data driven, and thematically. Top-down as well as bottom-up coding will be used. The top-down coding will use predefined codes based on factors mentioned in the theoretical models: the HALL framework, SPT, and RPM. The bottom-up coding (free coding) will trace general themes that emerge in interviews and focus groups. In this way, relevant topics devised in advance of the study design and relevant topics from practice will be fully mapped. These themes will make it possible to interrelate and interpret the gathered data [80].

Quantitative data will be analyzed by descriptive statistics and regression analysis techniques using the SPSS program. In the analysis, quantitative data obtained to measure changes in perceived health (RQ4) will be combined with qualitative data on participation, empowerment, and health literacy (RQ 2), with data at professional level – short-term outcomes realized by the healthy alliances and health brokers (RQ 3), and with data at community level – the social and the physical environment (RQ1).

The impacts on the different levels will be integrated and related to the action principles using realist synthesis [81] in the data analysis, facilitating the identification of the contextual factors and program mechanisms determining the outcomes (or impacts). These context–mechanism–outcome (CMO) configurations [18] will provide insights into the overall impacts in relation to the action principles.

### Sample size and power

The perceived health of adult inhabitants in the neighborhood will be used as the primary outcome of the VoM program at the individual level. In line with common practice in presenting perceived health prevalence rates, response options for self-perceived health will be dichotomized, with the response categories ‘very good’ and ‘good’ into one ‘very good or good’ category and the other response options in a ‘less than good’ category [82]. In 2015, the percentage of inhabitants in the city of Deventer scoring (very) good health was on average 79%, whereas this was 75% for the city district Voorstad [16]. Therefore, the estimate of the effect size of perceived health to be obtained by implementing the VoM program was determined by the difference between Voorstad (0.75) and the city of Deventer (0.79): 0.04. The sample size calculation was conducted with G*Power version 3.1.9.2. with alpha set on 0.05, and a power of 0.80. The used test family was exact and based on the difference from a constant (0.75). The required lower critical number of participants is 542, the required sample size is 697. The response rate of Health Monitors in general is 40% [83]. As there are differences in response rate between city districts, we assume a modest response rate of 35%. The required number of participants to obtain reliable estimates of increase in perceived health is therefore 2000. The total adult population in Voorstad is 8412 inhabitants. 2200 Inhabitants will be invited to join the online survey, in order to be sure of sufficient power.

## Discussion

### Relevance

This study will evaluate the impact of a community-based health program in a socioeconomically deprived city district in order to find keys to reducing health inequities. It is a single case study in which low SES inhabitants – in the view of health professionals usually hard to reach and not very interested in health promotion activities – are actively involved. It will provide insights into perceptions, values, and needs regarding the health of low SES groups.

The VoM program is innovative as it is different from usual health promotion programs in which health subjects and activities are set by professionals. Instead, the VoM program shifts from being a pre-devised health promotion program with a set of interventions to being an open approach with a focus on action elements. Unravelling the mechanisms of these action elements – citizen participation, intersectoral collaboration at community level, and a health supportive environment – will help to find ways to reduce health inequities. The findings will contribute to a better understanding, and will expand the knowledge, of what works for low SES groups and why it works. Other local health promotion programs can benefit from the knowledge and experiences gathered in this study.

### Strengths and limitations

The study design is optimized for internal and external validity because of the combination of action research, process evaluation, and citizens’ monitoring and survey data. The principle of triangulation is continuously applied to optimize the reliability of this study, using multiple methods and multiple sources. Internal validity is enhanced by triangulation of methods and resources, whereby results will be checked with other stakeholders. In addition, other verification techniques will be used, such as expert consultation.

In this study, the inhabitants’ survey will be used to measure perceived health and health determinants in a pre-test/post-test design. The results obtained from these surveys will be linked with results from the intervention, the environment, and the organizational level in order to be able to explain why changes in perceived health have taken place or not.

The application of SPT, the HALL framework, and RPM provides the researcher with a strong theoretical framework and guarantees validation of the results gathered in this single case study. This study contributes to the knowledge on the benefits of citizen participation, being a necessary aspect of health promotion, and how to realize it. Recent studies [84] recommend evaluation of community participation in creating a ‘health in all policies’ knowledge base. Hence, the participatory action research in itself contributes to health literacy, is empowering for those who participate, and contributes to community building [27].

## Abbreviations

CMO: Context–Mechanism–Outcome
HALL: Healthy Alliance
HLQ: Health Literacy Questionnaire
PNMT: Participatory Network Mapping Tool
RPM: Reasonable Person Model
RQ: Research Question
SES: Socioeconomic Status
SPT: Social Practice Theory
VoM: Voorstad on the Move
